# How does the brain remember? Distinct pathways for voluntary and involuntary recall

**DOI:** 10.1371/journal.pbio.3003328

**Published:** 2025-08-20

**Authors:** Xiaoqing Hu, Shengzi Zeng

**Affiliations:** 1 Department of Psychology, The State Key Laboratory of Brain and Cognitive Sciences, The University of Hong Kong, Pokfulam, Hong Kong SAR, China; 2 Center for Sleep and Cognition, Department of Psychiatry, Beth Israel Deaconess Medical Center, Boston, Massachusetts, United States of America; 3 Division of Sleep Medicine, Harvard Medical School, Boston, Massachusetts, United States of America

## Abstract

Involuntary memories come on spontaneously and can feel vivid and emotionally powerful. This Primer highlights a recent PLOS Biology study showing that involuntary and voluntary memory recall rely on distinct neural processes.

## Teaser

Memories can involuntarily come to mind out of nowhere. These spontaneous flashes often feel vivid and emotionally powerful. A new study in *PLOS Biology* reveals that involuntary and voluntary memory rely on distinct brain mechanisms.

Most of us have experienced involuntary memories, wherein we spontaneously recall an episode from the past. Sometimes, these memories are of a fond moment, such as a joyful birthday party. Other times, involuntary memories can stem from traumatic experiences, like a car crash during a traffic accident. Involuntary memories are more than just recollections of the event—they can bring back intense emotions like joy or fear [1]. In cases of trauma, involuntary memories can be particularly troubling. For individuals with post-traumatic stress disorder (PTSD), involuntary intrusion of traumatic memories is a central and distressing symptom, often triggering hyperarousal and avoidance behaviors [2,3].

Despite how common and clinically relevant these experiences are, scientists surprisingly know little about how the brain supports voluntary versus involuntary memories. The new research by Kobelt and colleagues, published in the current issue of *PLOS Biology,* offers important insights [4]. Using electroencephalography (EEG) to measure the brain’s electrical activity in real time via sensors on the scalp, the team demonstrated that involuntary and voluntary memory recall rely on different patterns of brain activity. In addition, they examined the brain rhythms such as the theta EEG power over the mid-frontal sensors to track cognitive interference [5,6]. This research not only deepens our understanding of how memory works but also has important implications for mental health. By elucidating why some memories—particularly traumatic ones—can feel so intrusive and difficult to control, it may inform the development of future treatments for conditions like PTSD.

In the experiment, participants were asked to learn pairs of abstract line drawings and common everyday objects, with the objects shown either on the left or right side of a computer screen. Later, their memory was tested in both voluntary and involuntary recall conditions. Specifically, during the voluntary recall, participants were asked to deliberately recall the object and its location when shown the abstract image. During the involuntary recall, participants performed a visual discrimination task using the same abstract cue, and were then asked whether any cue-associated object memories came to mind involuntarily. This setup allowed the researchers to compare brain activity during voluntary and involuntary memory retrieval.

**Fig 1:**
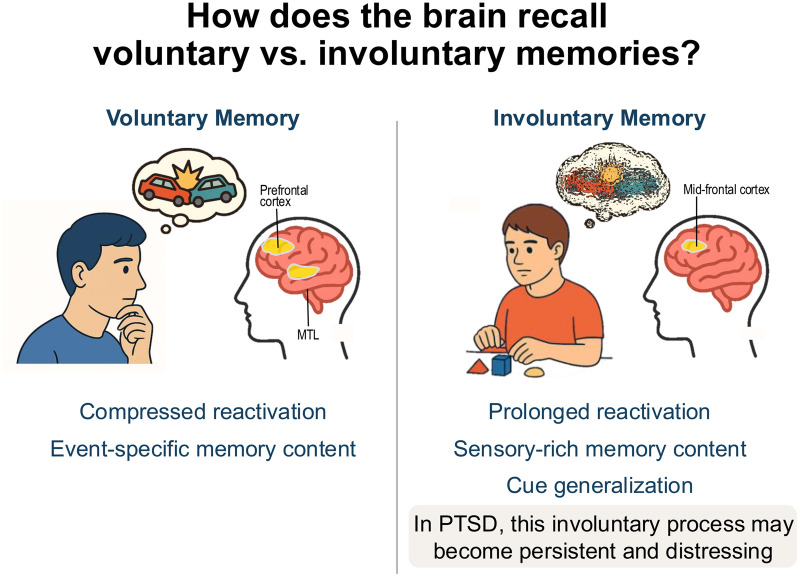
How does the brain recall voluntary vs. involuntary memory. During voluntary memory recall, participants actively retrieved item-specific details, engaging brain networks and EEG patterns associated with intentional memory retrieval. By contrast, involuntary recall—such as during memory intrusions—elicited prolonged reactivation of general sensory features rather than item-specific memory content. MTL: medial temporal lobe.

To unravel the hidden neural activity pattern of memory retrieval, the researchers employed the multivariate representation similarity analyses on EEG data. This analysis allows researchers to illuminate whether and how patterns of brain activity differ for voluntary versus involuntary memory. They found that involuntary recall is associated with a prolonged reactivation of general sensory features—such as which side of the screen the object originally appeared on—but did not bring back the specific details that uniquely defined that memory. By contrast, voluntary recall triggered compressed reactivation of item-specific patterns—suggesting that when we try to remember something intentionally, our brains zoom in on the unique elements that make a memory distinct (Fig 1).

Investigating neural oscillations, the results showed that involuntary retrieval was associated with a rapid increase in the mid-frontal theta EEG power, indicating enhanced cognitive interference. This helps explain why involuntary memory distracts us from ongoing tasks. By contrast, voluntary retrieval was associated with widespread theta EEG power across distributed scalp sensors, especially in the prefrontal cortex and the medial temporal lobe—brain regions that support deliberate memory recall.

Together, these findings suggest that involuntary and voluntary memories are not just two sides of the same coin. They differ in how they are retrieved, what kind of information they bring back, and how quickly they unfold in the brain. In particular, because they rely more on shared sensory features and less on item-specific details, these involuntary memories may be more easily triggered by a wide range of cues. They are also associated with temporally extended brain activity reflecting the prolonged reactivation of shared sensory features across similar cues. This sustained cue-generalized reactivation may help explain why involuntary intrusions often feel immersive and difficuly to control.

Although this study examines neutral memories, these results could shed light on intrusive memories in conditions like PTSD by informing the neural basis. For example, the study revealed that involuntary memory was associated with enhanced mid-frontal theta EEG power—a neural signature that has previously been linked to conflict detection and inhibitory processes during memory control [5]. This suggests that when an involuntary memory intrudes, the brain may perceive it as a conflict signal, triggering control-related neural systems in an attempt to manage the intrusion. In the context of PTSD, where intrusive memories are frequent and distressing, this pattern may reflect a heightened or dysfunctional conflict monitoring system that fails to regulate unwanted recalls. Understanding these neural dynamics would inform new psycho- or neuro-therapies aimed at helping individuals gain more control over unwanted memories. Future research would benefit from directly testing this mechanism in individuals with trauma histories.

By showing that voluntary and involuntary memory retrieval rely on distinct brain activities, it opens up new avenues for research and therapy. For example, prior studies have shown that engaging in visuospatially demanding tasks—such as playing *Tetris*—shortly after exposure to a lab-analogue traumatic event can reduce the frequency of intrusive memories [7]. This effect is thought to arise from interference with the sensory cortical representations that underlie involuntary memory [8]. Future studies could empirically test this proposed mechanism, clarifying how interventions operate and whether they generalize across different modalities or populations.

Furthermore, these findings raise the possibility of training individuals to exert greater control over involuntary memory processes. Could we train people to better control their involuntary memory, making them less frequent and intense [9,10]? With brain-based technologies advancing rapidly, could we one day develop real-time neuromodulation systems that detect the early signs of an intrusive memory and intervene to shorten its impact?

The study by Kobelt and colleagues offers solid foundations for these exciting questions. It reminds us that memory is not a single, unified function, but a rich, dynamic process. Whether we are deliberately recalling a precious moment or struggling with an unwanted involuntary memory, our brains navigate memory in ways that are far more nuanced and complex than we once thought.

## Abbreviations

EEG: electroencephalography
PTSD: post-traumatic stress disorder
